# Purification and characterization of midgut α-amylase in a predatory bug, *Andralus spinidens*

**DOI:** 10.1093/jis/14.1.65

**Published:** 2014-01-01

**Authors:** Sahar Sorkhabi-Abdolmaleki, Arash Zibaee, Hassan Hoda, Mahmoud FazeliDinan

**Affiliations:** 1 Department of Plant Protection, Faculty of Agricultural Sciences, University of Guilan, Rasht, Iran; 2 Biological Control Department, Iranian Research Institute of Plant Protection, Amol, Iran; 3 Department of Medical Entomology, Health Sciences Research Center, Faculty of Health, Mazandaran University of Medical Science, Sari, Iran

**Keywords:** amylolytic activity, biochemical property, inhibitor

## Abstract

α-Amylases are widespread enzymes that catalyze endohydrolysis of long α-1,4-glucan chains such as starch and glycogen. The highest amylolytic activity was found in 5th instar nymphs and midgut of the predatory bug, *Andrallus spinidens* F. (Hemiptera: Pentatomidae). The α-amylase was purified following a three-step procedure. The purified α-amylase had a specific activity of 13.46 U/mg protein, recovery of 4.21, purification fold of 13.87, and molecular weight of 21.3 kDa. The enzyme had optimal pH and temperature of 7 and 45°C, respectively. Na+, Mn+, Mg2+, and Zn2+ significantly decreased activity of the purified α-amylase, but some concentrations of K+, Ca2+, and Cu2+ had the opposite effect. EDTA, EGTA, and DTC significantly decreased enzymatic activity, showing the presence of metal ions in the catalytic site of the enzyme. Kinetic parameters of the purified α-amylase showed a Km of 3.71% in starch and 4.96% for glycogen, suggesting that the enzyme had a higher affinity for starch.

## Introduction

*Andrallus spinidens* F. (Hemiptera: Pentatomidae) is a predatory bug that has been considered as a biological control agent of caterpillars mainly in rice fields. The potential for *A. spinidens* to be used as a control agent has been reported for rice pests in India, Malaysia, and Iran ( Manley 1982 ; Mohaghegh and Najafi 2003 ). Both nymphs and adults feed on several caterpillars, such as *Chilo suppressalis* , *Naranga aenescens* , and *Helicoverpa armigera* , in the rice fields of northern Iran ( Mohaghegh and Najafi 2003 ). Najafi-Navaee et al. (1998) reported that *A. spinidens* in the rice fields of northern Iran has five generations per year.

Polysaccharides, like starch and glycogen, are the major components in cells and tissues of plants and animals, respectively. α-Amylases (EC 3.2.1.1) catalyze endohydrolysis of long α-1,4-glucan chains, such as starch and glycogen ( Terra and Ferriera 2005 ). Amylases extracted from insects are calcium-dependent and activated by chloride in their optimal pH ( Terra and Ferreira 1994 ). A comprehensive study ( Strobl et al. 1998 ) on larval digestive α-amylase of *Tenebrio molitor* L. (Coleoptera: Tenebrionidae) showed that the enzyme has three domains. The central domain (domain A) is an (b/a)8-barrel that comprises the core of the molecule and includes catalytic amino acid residues, and domains B and C are almost opposite to each other, on each side of domain A ( Strobl et al. 1998 ). Abundance and activity of insect α-amylases in the gut are dependent on food sources, so feeding on wool and plant tissues causes the lowest and highest amylolytic activity, respectively ( Chapman 1998 ; Zibaee et al. 2008 ).

*A. spinidens* is dependent on extra-oral or preoral digestion by using digestive enzymes secreted from salivary glands prior to final digestion in the midgut ( Zibaee et al. 2012b ). Extra-oral digestion is a process in which secreted enzymes from salivary glands are injected into the prey, thereby liquefying its solid tissues. This is a prevalent process in predaceous ground-dwelling arthropods and is known to occur in at least 38 of 62 families of heteropterans ( Cohen 1993 , 1995; Fialho et al. 2012 ). Ingested food is then digested further in the midgut. Food molecules, mainly polymers such as proteins and carbohydrates (starch or glycogen), are digested in three phases by α-amylase, exoand endopeptidases, glycosidases, and lipases ( Terra and Ferriera 2005 ).

In our previous studies, amylolytic, proteolytic, and lipolytic properties found in *A. spinidens* saliva were studied using purification and biochemical approaches ( Zibaee et al. 2011 , 2012a, b). In the case of salivary α-amylase, the molecular weight of the purified enzyme was 26 kDa, and the optimal pH and temperature were 9 and 35–40°C, respectively ( Zibaee et al. 2012a ). Kinetic parameters of the purified enzyme showed that both starch and glycogen are suitable substrates for enzymatic assay, but a lower Km demonstrated glycogen as a more appropriate substrate ( Zibaee et al. 2012a ). Also, using specific inhibitors, the presence of metal ions in the active site of the enzyme was verified ( Zibaee et al. 2012a ). Although ecological approaches (e.g., functional response) are a common way to find efficient biocontrol agents, digestive physiology may provide a better understanding of predator-prey interactions (PascualRuiz et al. 2009). Hence, it is important to examine digestive enzymes and their properties. The aim of the current study was the complete purification and characterization of a midgut α-amylase in *A. spinidens* .

## Materials and Methods

### *A. spinidens* rearing

A colony of *A. spinidens* was established by adults collected from harvested rice fields in Amol (Mazandaran, northern Iran), in late September 2011. Insects were reared on late instars of *Chilo suppressalis* L. (Lepidoptera: Crambidae) as prey and provided with wet cotton plugs fitted into small plastic dishes (2.5 cm diameter) as moisture sources at 25±1°C and 80% RH as laboratory conditions.

### Sample preparation

Insects were randomly selected, and the midguts were removed by dissection under a stereo microscope in icecold saline buffer (NaCl, 10 mM). The abdomen was opened and the midgut was separated from the other tissues, rinsed in icecold distilled water, placed in a pre-cooled homogenizer, and ground. Equal portions of the midgut and distilled water were used to ensure a desirable concentration of the enzymes. Homogenates were transferred to 1.5 mL centrifuge tubes and centrifuged at 13000 rpm for 15 min at 4°C. The supernatants were pooled and stored at −20°C for subsequent analyses.

### α-amylase assay

The α-amylase activity was assayed using the dinitrosalicylic acid procedure ( Bernfeld 1955 ), using 1% soluble starch (Merck, ( www.merck.com ) as the substrate. 20 µL of the enzyme was incubated for 30 min at 35°C with 80 µL of phosphate buffer (0.02 M, pH 7.1) and 40 µL of soluble starch (1%). The reaction was stopped by addition of 100 µL dinitrosalicylic acid and heated in boiling water for 10 min prior to read absorbance at 545 nm. One unit of α-amylase activity was defined as the amount of enzyme required to produce 1 mg maltose in 30 min at 35°C. The negative control contained all reaction mixtures, but the enzyme was pre-boiled for 15 min to remove the enzymes in the samples. All assays were performed in duplicate, and each assay was repeated at least three times.

### Purification process

The purification process of midgut α-amylase in *A. spinidens* was carried out based on Englard and Seifter (1990) and Dennison (1999) procedures. The crude extract (40 mL) from midgut homogenates of *A. spinidens* adults was treated with ammonium sulfate at 4°C to give fractions precipitated at 35% and 75% saturations. The precipitates were collected by centrifugation at 6000 rpm for 15 min, diluted in 2 mL of Tris-HCl (20 mM, pH 8.8), and dialyzed overnight at 4°C against the same buffer. The enzyme solution was applied to a Sepharyl G-100 column, equilibrated with the same buffer. The column was run at a flow rate of 0.5 mL/min. Amylase activity was measured as described above. Fractions containing higher enzymatic activity were pooled and applied to a diethylaminoethyl (DEAE)- cellulose column, equilibrated with Tris-HCl (pH 8.8). The enzyme was eluted at a flow rate of 0.5 mL/min with a linear NaCl gradient (0–0.6 mol). Fractions (1.5 mL/tube) were collected, and their protein concentration and α-amylase activity were determined as previously described ( Bernfeld 1955 ; Bradford 1976 ). In the final step, fractions containing the highest enzymatic activity were pooled and used as the enzyme source.

### Sodium dodecylsulphate polyacrylamide gel electrophoresis (SDS-PAGE)

SDS-PAGE was performed according to the procedure described by Laemmli (1970) . The acrylamide concentration was 10% for the separating gel and 4% for the stacking gel. After running the gel at 100 mV as constant voltage, proteins on the polyacrylamide gel were stained with 0.2% Coomassie brilliant blue R-250 (Hames 1998). α-galactosidase (116 kDa), bovine serum albumin (66.2 kDa), ovalbumin (45 kDa), lactate dehydrogenase (35.5 kDa), restriction endonuclease bsp 981 (25 kDa), b-lactoglobulin (18.4 kDa), and lysozyme (14.4 kDa) were used as molecular mass standards.

### Effect of pH on enzyme activity

The pH optima of amylase were determined using Tris-HCl (20 mM) buffer. The tested pH levels were 3, 4, 5, 6, 7, 8, 9, 10, 11, and 12. The enzymatic assay was done as described above.

### Effect of temperature on enzyme activity

The effect of temperature on amylolytic activity was determined by incubating the reaction mixture at the following temperatures for 30 min: 10, 15, 20, 25, 30, 35, 40, 45, 50, 55, 60, 70, and 80°C.

### Effect of mono- and divalent cations on α- amylase activity

The effects of various cations on purified α- amylase activity were investigated using CaCl2, Mg Cl2, NaCl, KCl, CuSO4, and ZnSO4. In each case, 10 µL of a solution containing a concentration of ions (0, 0.5, 1, 3, 5, and 10 mM) and 10 µL of enzyme were preincubated for 10 min at pH 7 of universal buffer and 30°C as optimal temperature. 30 µL of starch was added to the mixture, and incubation was continued. The reaction was stopped by addition of 100 µL dinitrosalicylic acid and heated in boiling water for 10 min prior to read absorbance at 545 nm.

### Effect of specific inhibitors on α-amylase activity

The effects of enzyme inhibitors on α-amylase activity were studied using different concentrations (0, 0.5, 1, 3, 5, and 10 mM) of ethylene glycol-bis (β-aminoethylether) N, N, N′, N′-tetraacetic acid (EGTA), triethylenetetramine hexaacetic acid (TTHA), ethylenediamine tetraacetic acid (EDTA) and diethyldithiocarbamate (DETC). The purified enzyme (10 µL) was preincubated for 10 min at pH 7 and 30°C with 10 µL of inhibitors (at given concentrations). 50 µL of starch was added to the mixture, and the experiment was continued as earlier. The reaction was stopped by addition of 100 µL dinitrosalicylic acid and heated in boiling water for 10 min prior to read absorbance at 545 nm.

### Kinetic studies

Kinetic parameters of the purified α-amylase were calculated by using different concentrations (0.1, 0.2, 0.4, 0.6, 0.8, and 1%) of starch and glycogen. 10 µL of enzyme was preincubated for 10 min at pH 7 of Tris-HCl and 30°C as the optimal temperature. 30 µL each of starch and glycogen (different concentrations) were added to the mixture, and incubation was continued. Obtained data were inserted in Sigma Plot software (Version 6, ( www.sigmaplot.com ), and line regression was drawn by used concentrations of substrate and observed enzymatic velocity.

### Protein determination

Protein concentration was determined either by measuring absorbance at 545 nm by a kit provided by Ziest Chem ( www.zeistchem.com ) or by the method of Bradford (1976) using bovine serum albumin as standard.

### Statistical analysis

All data were compared by oneway analysis of variance (ANOVA) followed by Tukey’s studentized range distribution when significant differences were found at *P* ≤ 0.05. Differences between samples were considered statistically significant at *P* < 0.05 and marked in figures and tables with letters and asterisks.

## Results

**Figure 1.**
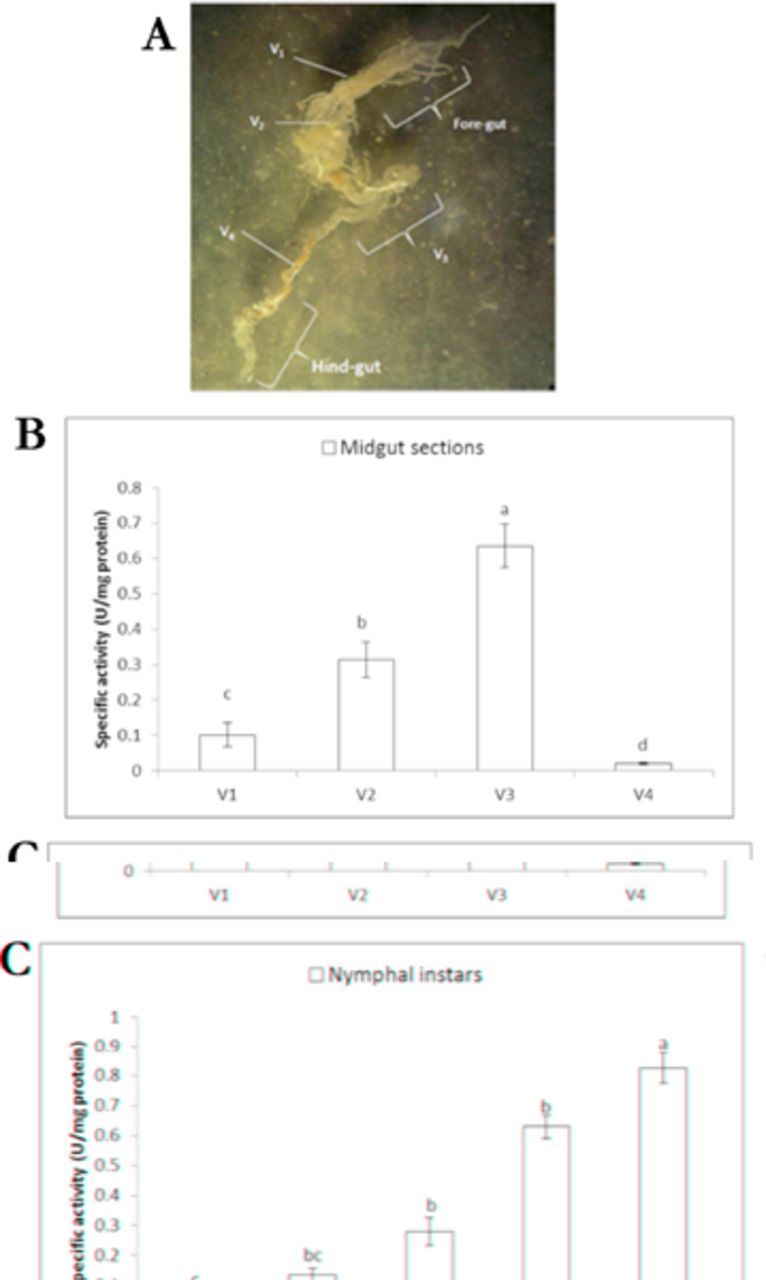
Amylolytic activity in the different midgut sections (a, b) and nymphal instars (c) of *Andrallus spinidens* . Statistical differences have been shown by different letters (Tukey’s test, *P* ≤ 0.05). High quality figures are available online.

Amylolytic activity was found in different nymphal instars and sections of the midgut sections (ventriculus; V1 to V4). The highest amylolytic activity was found in V3 while the lowest one was in V4 ( Figure 1a , b). Amylolytic activity increased in each of the nymphal instars so that the lowest and the highest activities were found in 1st and 5th instar larvae, respectively ( Figure 1c ). The lower amylolytic activity in the 1st nymphal instar could be attributed to the smaller amount of food ingested, which increases during nymphal development.

**Figure 2.**
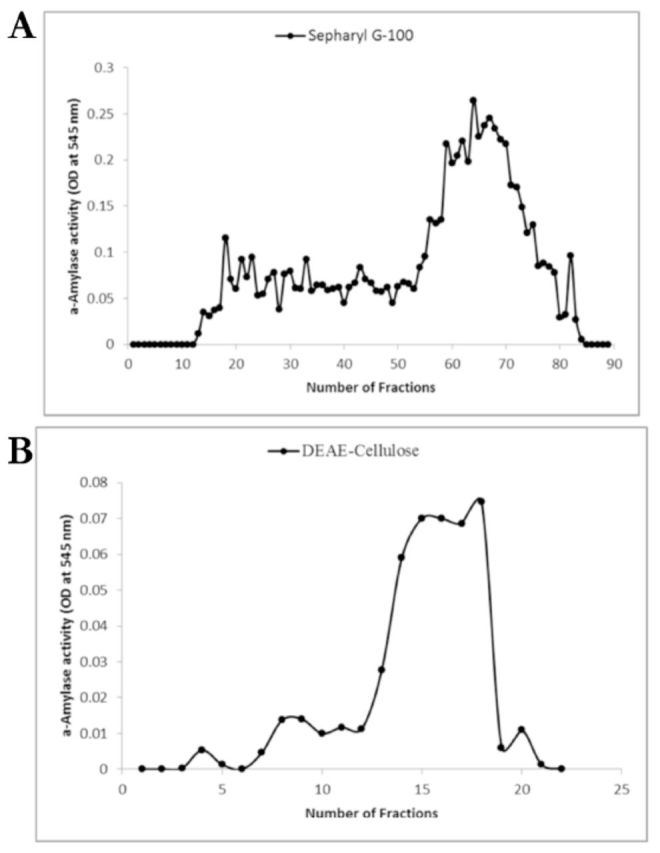
Column chromatography of the midgut α-amylase in the adult *Andrallus spinidens* . a) Sephacryl G-100 gel-filtration of the lipase after ammonium sulfate (35% and 75%) treatment. Fractions 26–37 contained the highest enzymatic activity with starch 1% and were collected for the next step. b) DEAE-cellulose ion-exchange chromatography of the gel-filtration samples. Fractions containing the highest enzymatic activity were used for characterization experiments. High quality figures are available online.

The midgut α-amylase of *A. spinidens* was purified following a three-step procedure. Samples from homogenized midguts were precipitated by 35% and 75% concentrations of ammonium sulfate. Samples from the 35% and 75% ammonium sulfate precipitation were dialyzed against the same buffer (Tris- HCl, 20 mM) for 20 hours to remove ammonium sulfate. The dialyzed sample was loaded on a Sepharyl G-100 column ( Figure 2a ). Fractions 59–70 had the highest amylolyic activity, containing 0.12 mg/dl protein with total enzymatic activity of 0.35 U ( Table 1 ). These fractions were pooled and loaded on the DEAE-cellulose column for ion exchange chromatography ( Figure 2b ). Using a NaCl gradient of 0–0.5 M, fractions were eluted, and fractions 14–18 had high enzymatic activities ( Figure 2b ). These fractions were pooled and had a protein concentration of 0.012 mg/dl protein and a total enzymatic activity of 0.16 U ( Table 1 ). The final purification step achieved 13.87-fold purity with a recovery of 4.21% and a specific activity of 13.46 U/mg protein ( Table 1 ). SDS-PAGE was used to follow the progress of purification. SDS- PAGE revealed a protein with 21.3 kDa of molecular mass.

**Figure 3.**
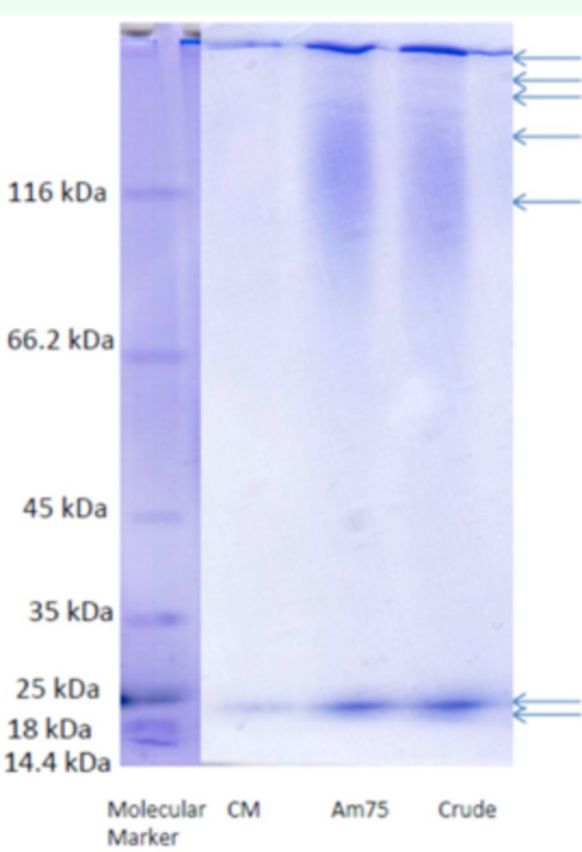
SDS- PAGE showing purity of midgut α-amylase in the adult *Andrallus spinidens* . High quality figures are available online.

**Table 1.**
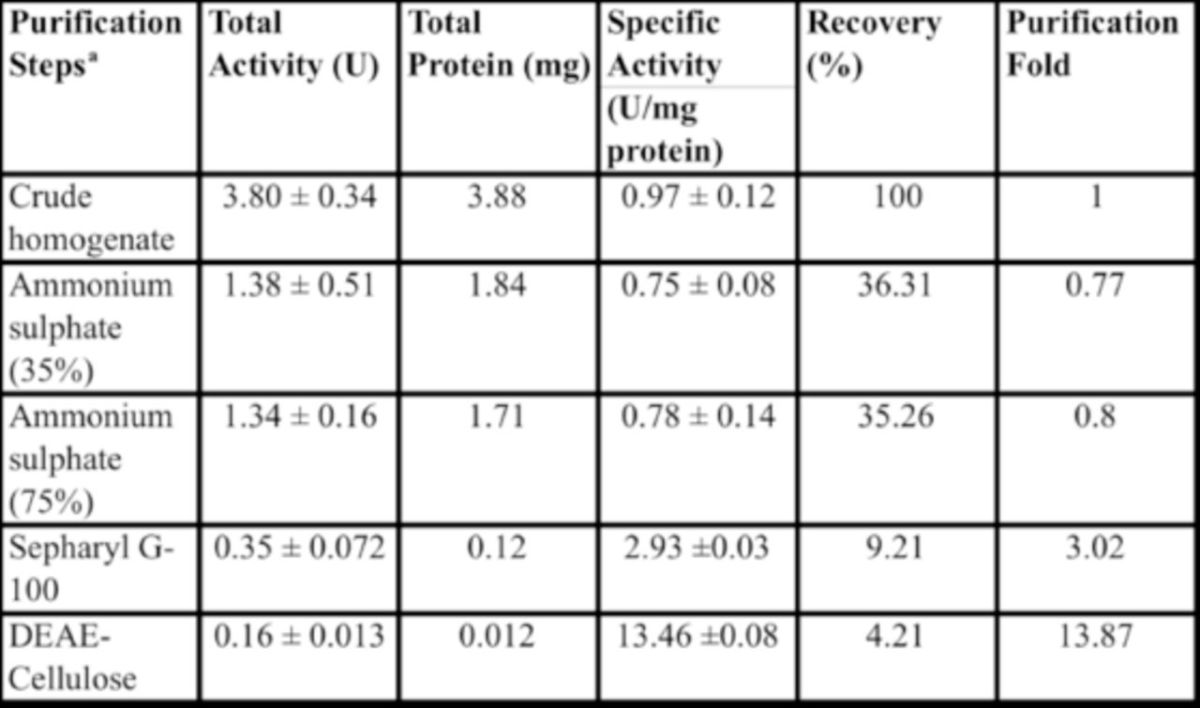
Purification of the midgut α-amylase from adult *Andrallus spinidens* .

aAfter precipitation of the crude homogenate by 35 and 75% of ammonium sulfate, samples were transferred to gel-filtration on a Sephadex G-100 column. Fractions with the highest lipase activity were pooled and loaded to DEAE-Cellulose column for ion exchange chromatography.

**Figure 4.**
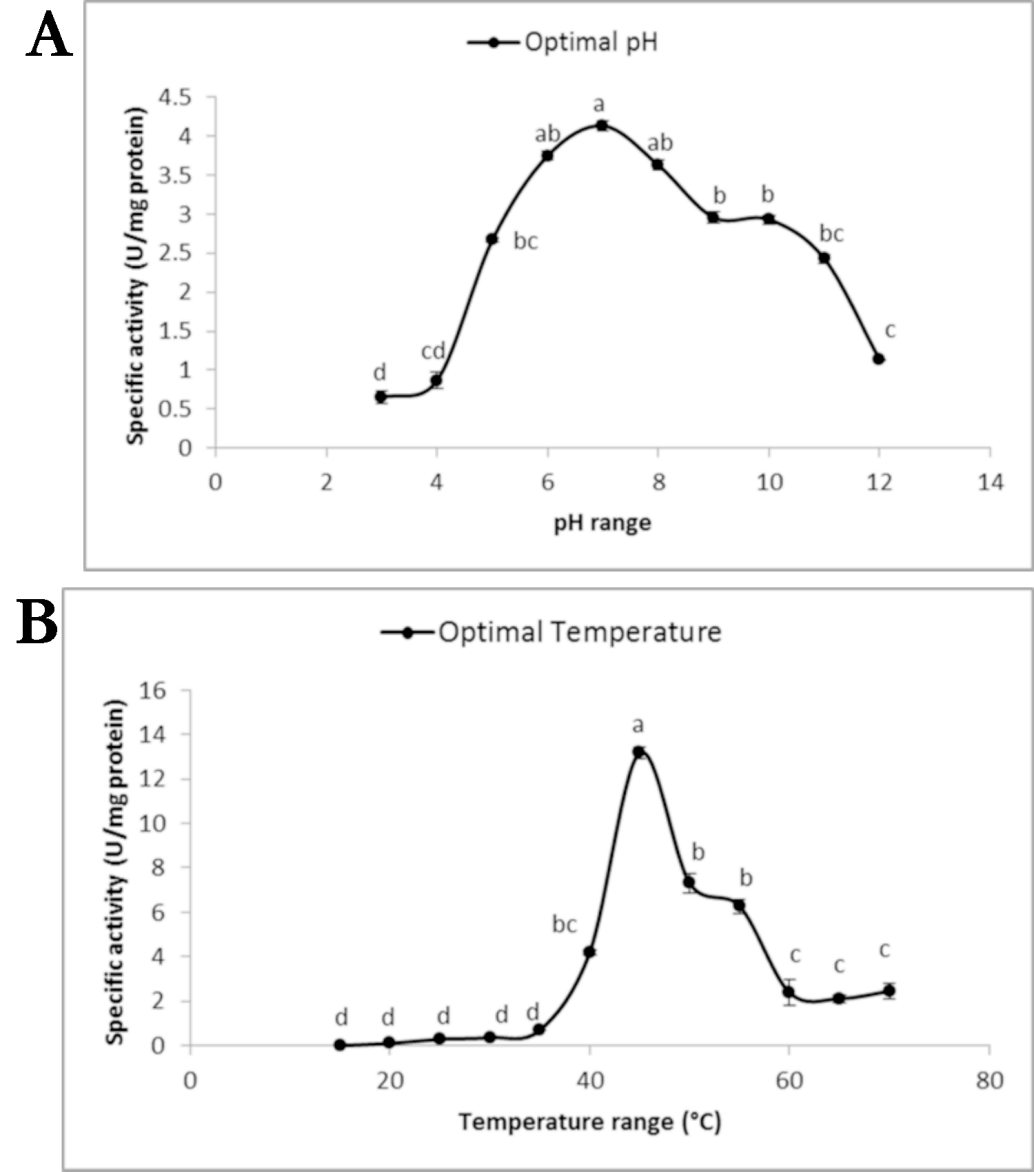
Optimal pH (A) and temperature (B) determination of the purified midgut α-amylase of the adult *Andrallus spinidens* . Statistical differences have been shown by different letters (Tukey’s test, *P* ≤ 0.05). High quality figures are available online.

In the current study, the optimal pH of midgut α-amylase was between pH 6 and 8, and the optimal temperature was 45°C, ( Figure 4a , b). In contrast, the salivary α-amylase had an optimal pH of 9 and an optimal temperature of 40°C, ( Zibaee et al. 2012a ).

**Figure 5.**
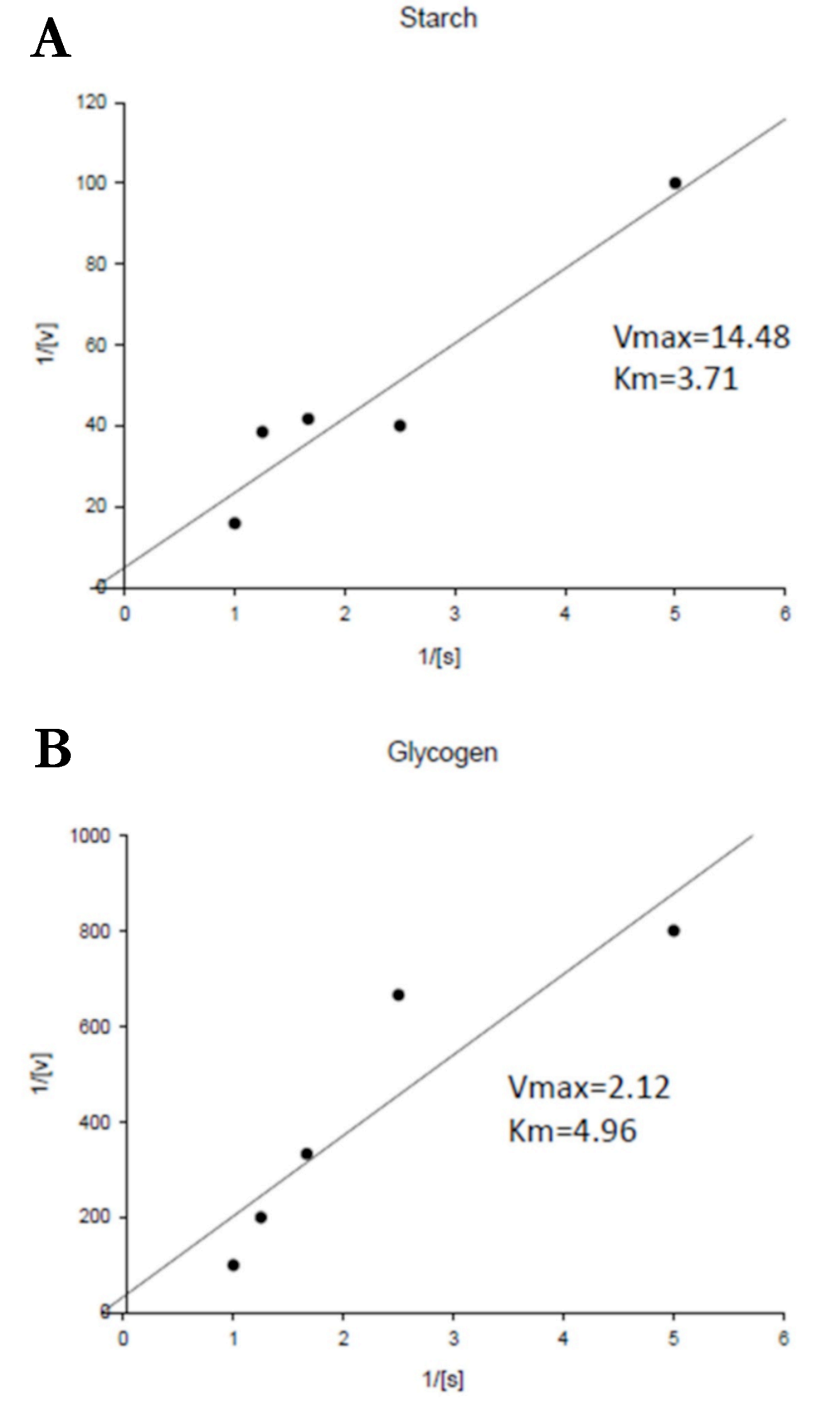
Double reciprocal plot to show the kinetic parameters of the purified midgut α-amylase of adult *Andrallus spinidens* by using starch and glycogen as substrates (1/Vmax = intercept on the 1/V0 ordinate, -1/Km = intercept on the negative side of the 1/[S] abscissa). High quality figures are available online.

Different concentrations of ions and their specific chelating agents significantly affected activity of the purified α-amylase in *A. spinidens* . Different concentrations of Na+, Mn+, Mg2+, and Zn2+ significantly decreased enzymatic activity up to 12% ( Table 2 ). Concentration of 3 mM of K+ increased the enzymatic activity, but other concentrations had no effect ( Table 2 ). A concentration of 0.5 mM of Ca2+ and concentrations of 3 and 5 mM of Cu2+ significantly increased activity of the purified α-amylase ( Table 2 ). In the case of specific inhibitors, EDTA as general chelating agent, EGTA as calcium chelating agent and DTC as cooper chelating agent significantly decreased enzymatic activity ( Table 3 ).

**Table 2.**
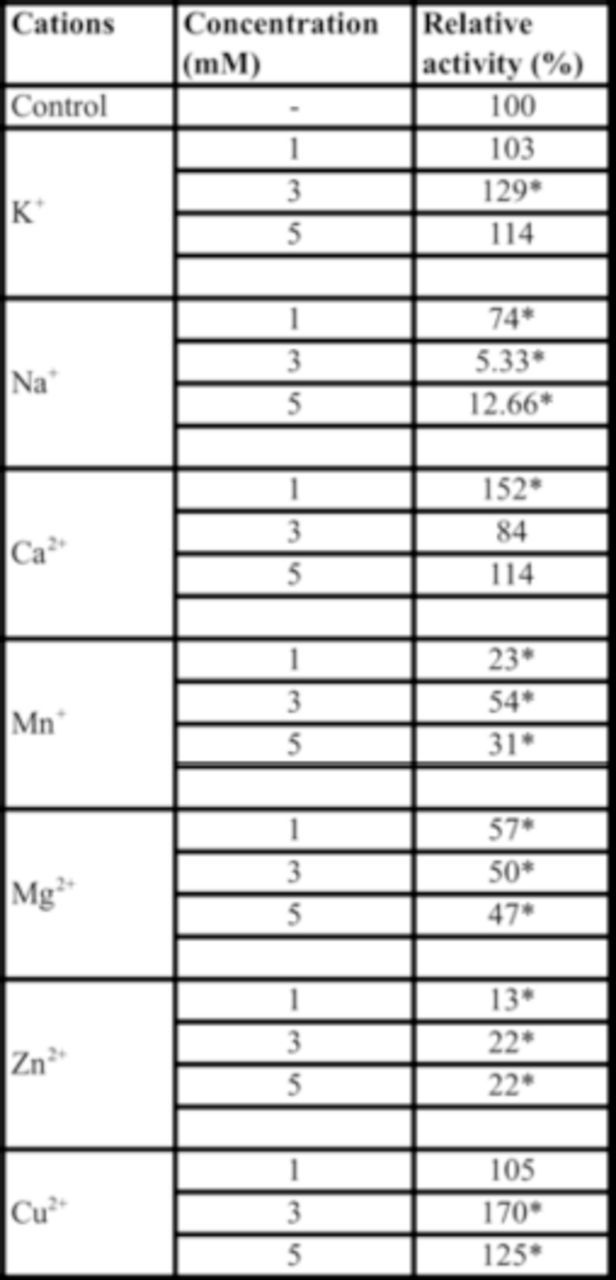
Effect of monovalent and divalent cations on purified midgut α-amylase of adult *Andrallus spinidens* .

All experiments were conducted at pH 7 and 30°C. Asterisks show statistical differences among used concentrations of cations and control (Tukey’ test, *P* ≤ 0.05).

**Table 3.**
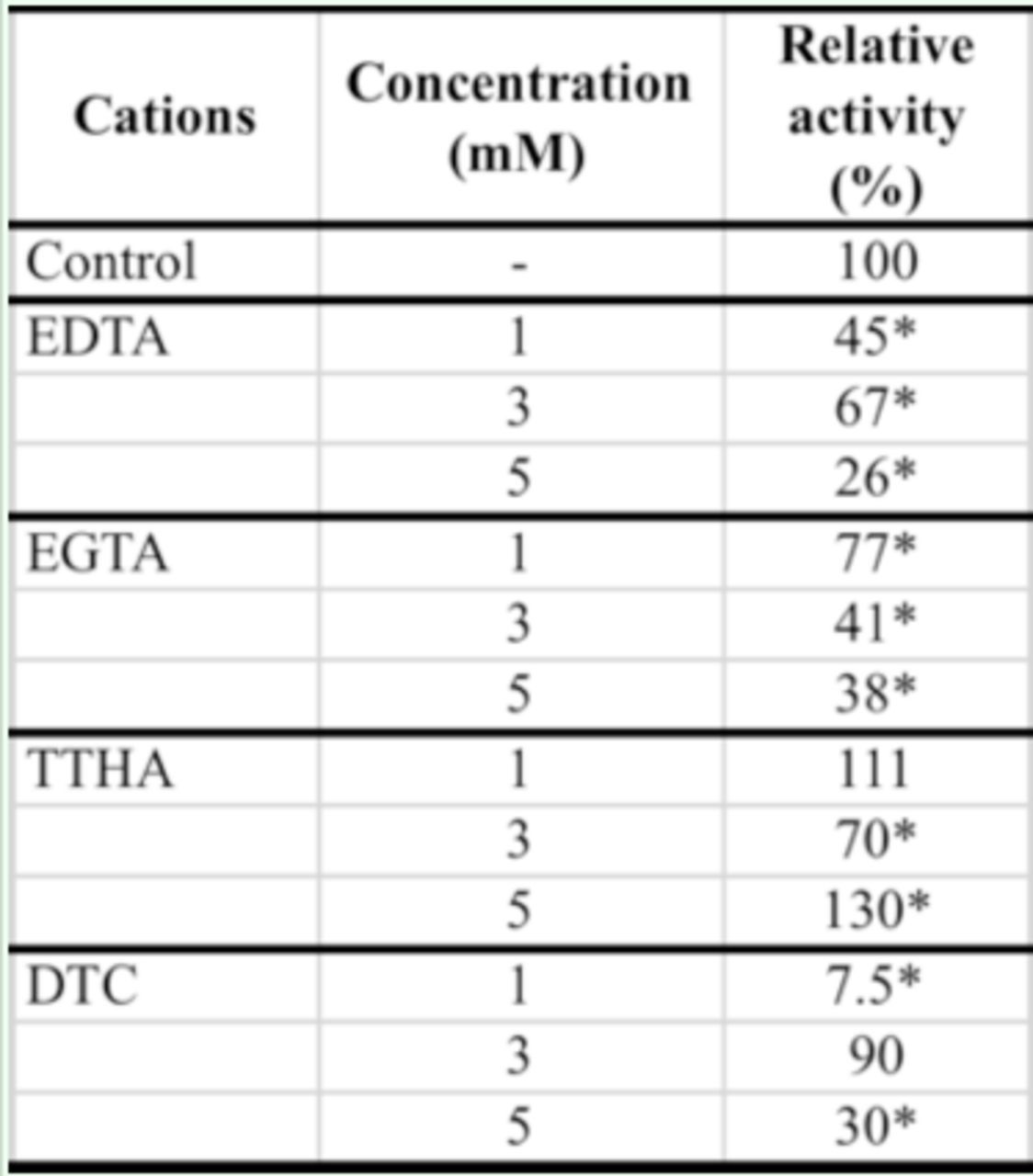
Effect of specific inhibitors on purified midgut α- amylase of adult *Andrallus spinidens* .

All experiments were conducted at pH 7 and 30°C. Asterisks show statistical differences among used concentrations of cations and control (Tukey’ test, *P* ≤ 0.05).

Kinetic parameters of the purified α-amylase of *A. spinidens* using Lineweaver-Burk analysis showed a Km of 3.71% when expressed in percentage of substrate (starch) and 4.96% for glycogen. As the Km value indicates enzyme- substrate specificity, it seems that the purified α-amylase demonstrates a higher affinity for starch, a phenomenon that has been reported for *Drosophila* species and *Pyrococcus furiosus* ( Prigent et al. 1998 ; Yang et al. 2004 ).

## Discussion

A major characteristic of the hemipteran alimentary canal is that the midgut has four sections, usually called V1, V2, V3, and V4 ( Saxena 1963 ). During diapause, hemipterans rely on food stored in the first midgut region (V1), which is large and sac-like ( Saxena, 1954 ). The role of V2 is to connect the main storage section (V1) with the digestion and absorption site (V3) ( Saxena 1954 ; Goodchild 1966 ). Symbionts accumulate in region V4 in *Nezara viridula* and possibly in other hemipterans ( Chapman 1998 ), including *Eurygaster integriceps* ( Goodchild 1966 ; Hirose 2005). Mehrabadi et al. (2009) found that the midgut enzymes, including amylases, glycosidases, and proteases, were found in region V3 of *E. integriceps* . The detailed structure of the ventricular regions has been documented in studies of *E. integriceps* ( Mehrabadi et al. 2012 ). Microvilli and perimicrovillar membranes were found in V1–V3 regions, with columnar cells characterized by presence of mitochondria, rough endoplasmic reticulum, and basal infoldings in the basal portion. The V4 region showed different histological features from the other three midgut regions, as it had a vacuolated epithelium with crypts that stored symbiotic bacteria. These results suggest that V1–V3 are involved in enzymatic activity and absorption, whereas V4 probably has no function in digestion ( Mehrabadi et al. 2012 ).

The normal molecular masses of α-amylases in insects vary from 28 to 87 kDa ( Terra and Ferreira 1994 ), but some exceptions have been found, including 132 kDa for *Orius insidiosus* ( Zeng and Cohen 2000b ) and 103 kDa for *Lutzomyia longipalpis* ( Vale et al. 2012 ). In the current study, a large smear of proteins with at least seven bands were present in the crude extract, but a single major protein band with a molecular mass of 21.3 kDa was present after ion exchange. In comparison, the salivary α-amylase has a molecular mass of 26 kDa ( Zibaee et al. 2012a ). Some amylases have three domains, one of which can be lost due to the activity of proteinases (personal communication, Prof. Octavio L. Franco, Universidade Catolica de Brasilia). Given the presence of many digestive proteases in the midgut of *A. spinidens* (Sorkhabi et al., personal observation), the low molecular weight of purified α-amylase may reflect the effect of digestive enzymes during extraction.

Temperature and pH are the two crucial factors that affect enzymatic activity. These are especially important as insects are poikilothermic organisms and the physiology of their body is directly influenced by environmental temperature. The optimal pH for α-amylases of several coleopteran insects was determined to be 5.2–5.4 ( Baker 1983 ; Baker 1991 ). The optimal pH for α-amylases of two hemipteran species was 6.5 (Zeng and Cohen 2000), which is near to our finding for *A. spinidens* . Bandani et al. (2005) found an optimal pH of 6.5 and an optimal temperature of 25–40°C for purified α-amylase from the midgut of *E. integriceps* . Pelegrini et al. (2006) found optimal temperatures of 20°C and 30°C for the purified α-amylase for the beetle *Zabrotes subfasciatus;* the activity of the enzyme sharply decreased when the temperature reached 60°C. This decrease of the purified enzyme activity might be attributed to denaturation of hydrophobic residues on the surface of the enzyme that are important for α-amylase stabilization, such as tryptophan and phenylalanine ( Pelegrini et al. 2006 ). The high activity of an enzyme at a specific temperature *in vitro* reflects the effects of environmental temperature where the organism lives and feeds on the hosts. Our results may reflect the high environmental temperatures of rice fields in northern Iran, which can be between 30 and 45°C in June through September ( Zibaee et al. 2012b ).

In general, midgut α-amylases require calcium for their maximum catalytic activity. Calcium also affords stability for the amylases in a variety of animals, including insects (Kazzazi et al. 2009). Pelegrini et al. (2006) found that a low concentration of calcium increased amylolytic activity in *Z. subfaciatus* (like in *A. spinidens* ). Inhibition of purified midgut α- amylase activity by EGTA confirms the presence of calcium ion in the catalytic site of the enzyme. The increase in enzyme activity by Cu2+ and its inhibition by DTC could be attributed to replacement of Cu2+ by Ca2+ in the active site of the enzyme because of their chemical polarity. Meanwhile, decreasing activity of the purified midgut α-amylase in the presence of some other ions could be attributed to binding between the inhibitor and enzyme or by ion chelation ( Pelegrini et al. 2006 ). It seems this inhibition causes calcium replacement, as observed in α-amylases from mammalian serum ( Yamasaki 2003 ).

In summary, the presence of a low weight of midgut α-amylase was verified in *A. spinidens* . Because glycogen is the main storage polysaccharide in animals’ bodies, midgut amylases might have a crucial role in digestion of prey tissues. Hence, a detailed biochemical and molecular analysis of midgut amylase upon exposure of the insect to a range of preys will highlight importance of amylases in digestion of *A. spinidens* .
